# Improving 24-Hour Medication Reconciliation Through Audit and Feedback: A Multicycle Quality Improvement Study at a Scottish District General Hospital

**DOI:** 10.7759/cureus.84445

**Published:** 2025-05-20

**Authors:** William Stanley, Kate Falconer, Megan Hume

**Affiliations:** 1 Emergency Medicine, Queensland Health, Rockhampton, AUS; 2 General Medicine, Gloucestershire Hospitals NHS Foundation Trust, Gloucester, GBR; 3 General Medicine, NHS Borders, Melrose, GBR

**Keywords:** audit and feedback, clinical audit, documentation compliance, hospital admissions, medication reconciliation, multidisciplinary care team, patient safety, prescribing safety, quality improvement, withheld medications

## Abstract

Introduction

Medication reconciliation (MR) is a key patient safety process during hospital admission. MR ensures an accurate list of a patient’s medications is compiled on hospital admission, enabling safe downstream prescribing and reducing adverse events. This responsibility falls primarily to the admitting clinician in paper-based systems with limited pharmacy input at the point of admission. This study aimed to assess local compliance with the national benchmarks and evaluate whether a structured audit-feedback intervention could improve MR documentation and the recording of reasons for withholding medications.

Methods

This quality improvement project was conducted over four 1-week periods: an initial audit, a pre-intervention baseline, a post-intervention follow-up, and a reaudit 10 days later. The medical records and prescription charts of 305 acute admissions were reviewed within 24 hours of presentation. The primary process measures were (i) the proportion of patients with a documented MR and (ii) the proportion of withheld medications with a documented reason, recorded in the clinical notes, drug chart, or MR sheet. The intervention included personalized compliance feedback to admitting doctors and consultants, public recognition of high performers, and educational posters placed in clinical areas. Two-proportion Z-tests were used to assess significance.

Results

The proportion of patients with a documented MR increased from 55 of 73 (75.3%) pre-intervention to 50 of 65 (89.3%) post-intervention (p = 0.044), and further to 64 of 69 (92.8%) at reaudit (p = 0.0049). The proportion of withheld medications with a documented reason rose from 23 of 39 (59.0%) to 30 of 37 (81.1%) post-intervention (p = 0.036) and remained elevated at 35 of 44 (79.6%) during the reaudit (p = 0.041). The most commonly withheld medication classes were diuretics (n = 27, 14.5%), angiotensin-converting enzyme inhibitors or angiotensin receptor blockers (n = 24, 12.9%), and statins (n = 16, 8.6%). The most frequent reasons for withholding were acute kidney injury or dehydration (n = 39, 27.8%), hypotension (n = 15, 11.5%), and medications deemed not indicated (n = 15, 11.5%).

Conclusion

A targeted, low-cost audit-feedback intervention directed at admitting clinicians significantly improved compliance with MR standards and the documentation of withheld medications. These findings suggest that, even in resource-limited, paper-based settings, behavioral strategies can deliver meaningful improvements in prescribing safety and move practice closer to national standards.

## Introduction

Medication reconciliation (MR) is a key component of the hospital admission process. The National Institute for Health and Care Excellence (NICE) defines MR as the process of identifying an accurate list of a patient’s current medications and comparing it with the list prescribed during their hospital stay [1]. There is a well-recognized risk of prescribing errors at the point of admission, which can lead to patient harm and increased healthcare costs [2-5]. The Scottish Patient Safety Programme (SPSP) recommends that 95% of patients should have a documented MR within 24 hours of admission [6].

Pharmacist-led MR programs have been shown to reduce medication discrepancies [7]. However, both the World Health Organization’s High 5s Project and NICE emphasize the importance of a multidisciplinary approach to MR [1,8]. In practice, the admitting clinician is typically responsible for prescribing medications during the initial admission period, prior to pharmacist review [9]. As such, a specific audit-feedback intervention targeting admitting clinicians may lead to measurable improvements in MR and prescribing safety.

In a paper-based acute medical unit of a Scottish district general hospital, admitting clinicians are responsible for performing MR during the admission process. Regular medications are either prescribed or withheld, and reasons for withholding should be clearly documented. Although MR may be reviewed later by a pharmacist, the volume of admissions and discharges means that review is typically prioritized for patients with complex or comorbid conditions.

This study aimed to assess compliance with the SPSP benchmark and to improve both the proportion of patients with documented MR and the clarity of documentation regarding withheld medications.

## Materials and methods

Process measures 

This study had two primary process measures: (i) the proportion of patients who had a documented MR completed by the admitting doctor within 24 hours of hospital admission and (ii) the proportion of withheld medications with a documented reason for withholding, recorded either in the clinical notes, on the drug chart, or on the medication reconciliation sheet.

Data collection

Data were collected prospectively by the audit team during four discrete one-week periods: an initial audit week, a pre-intervention week, a post-intervention week, and a reaudit week. Across these periods, 305 patient admissions to the acute medical unit at the Borders General Hospital, Scotland, were reviewed. Documentation was assessed within the first 24 hours of admission using the patients’ paper-based clinical records and prescription charts. Selection of patients was random, and there were no exclusion criteria. 

The initial audit week was used to identify areas requiring improvement and informed the selection of process measures. The pre-intervention week established a baseline for comparison and also served as the basis for designing a targeted intervention. Data analysis involved comparing the process measures from the pre-intervention period with those from the post-intervention and reaudit periods. Statistical significance was evaluated using two-proportion Z-tests.

Additional data were collected for each patient regarding the class of withheld medications and the reasons documented for withholding medications. 

Intervention

The intervention was devised using data from the pre-intervention week. For each patient, the names of the admitting clinician and post-taking consultant were recorded alongside MR and medication withholding data. Individual compliance rates were calculated for each admitting doctor, including the proportion of patients with completed MR and the proportion of withheld medications with a documented reason.

Each admitting clinician and consultant received a personalized email outlining their compliance rates, benchmarked against anonymized group averages. To reinforce positive behavior, the names of the highest-performing clinicians were displayed on a noticeboard in the doctors' office. Educational posters highlighting the importance of timely MR and documentation of withheld medications were placed in key clinical areas, including the emergency department and doctors' offices.

The intervention was implemented following the pre-intervention week. The post-intervention week immediately followed and served to assess the short-term impact of the measures. A second round of feedback was then provided using post-intervention data. This second round of individualized and group feedback was delivered in the same way as the first. A final reaudit was conducted 10 days later to evaluate whether improvements were sustained.

## Results

Primary process measures 

*Documented Medication R* *econciliation*

**Figure 1:**
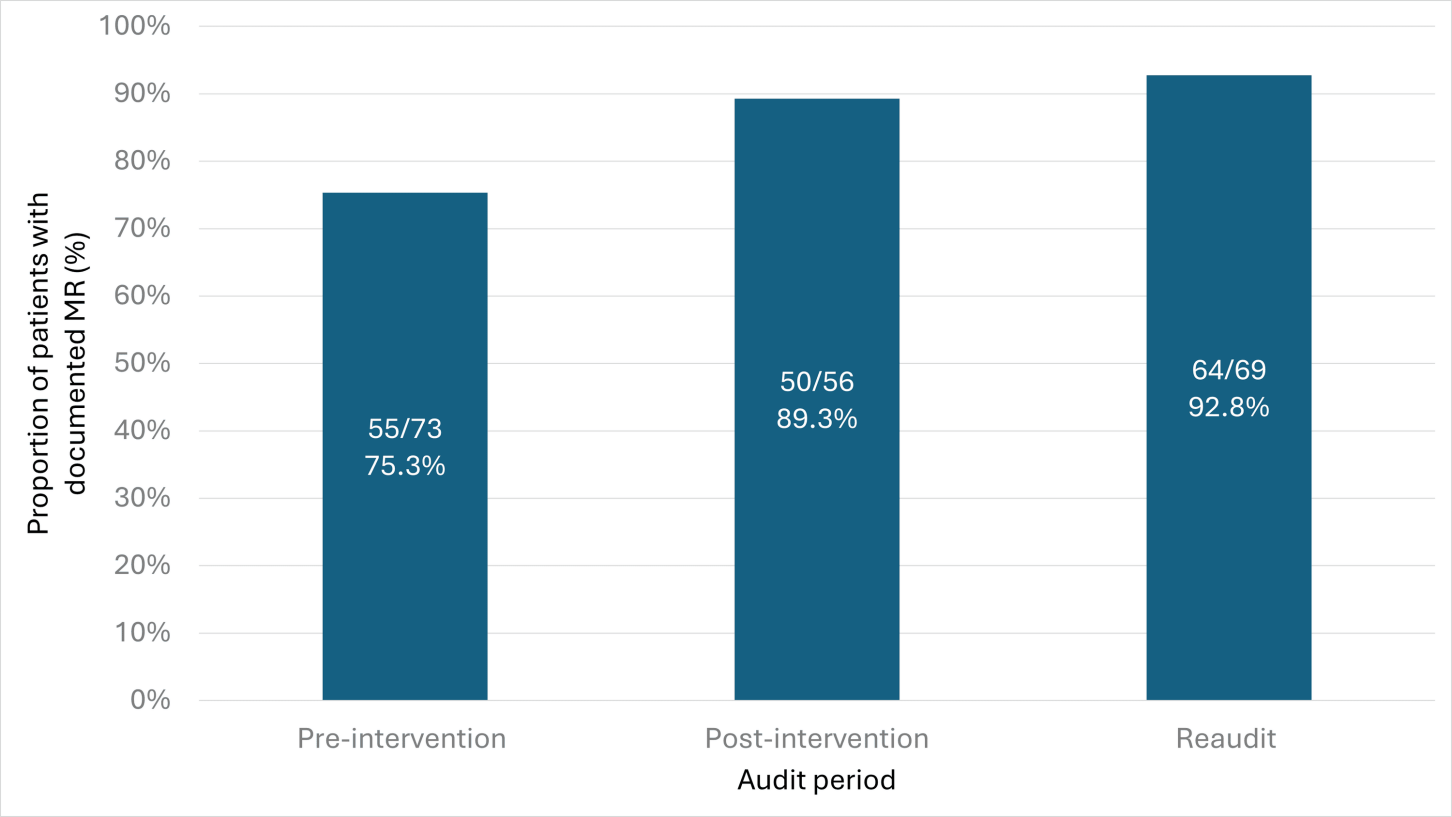
Proportion of patients with documented medication reconciliation (MR) within 24 hours of admission across the pre-intervention, post-intervention, and reaudit periods

There was a statistically significant improvement in the proportion of patients with a documented MR within 24 hours of admission following the intervention (Figure 1). Compliance increased from 55 of 73 (75.3%) pre-intervention to 50 of 56 (89.3%) post-intervention (p = 0.044), and further to 64 of 69 (92.8%) at reaudit (p = 0.0049).

Documented Reason for Withholding

**Figure 2:**
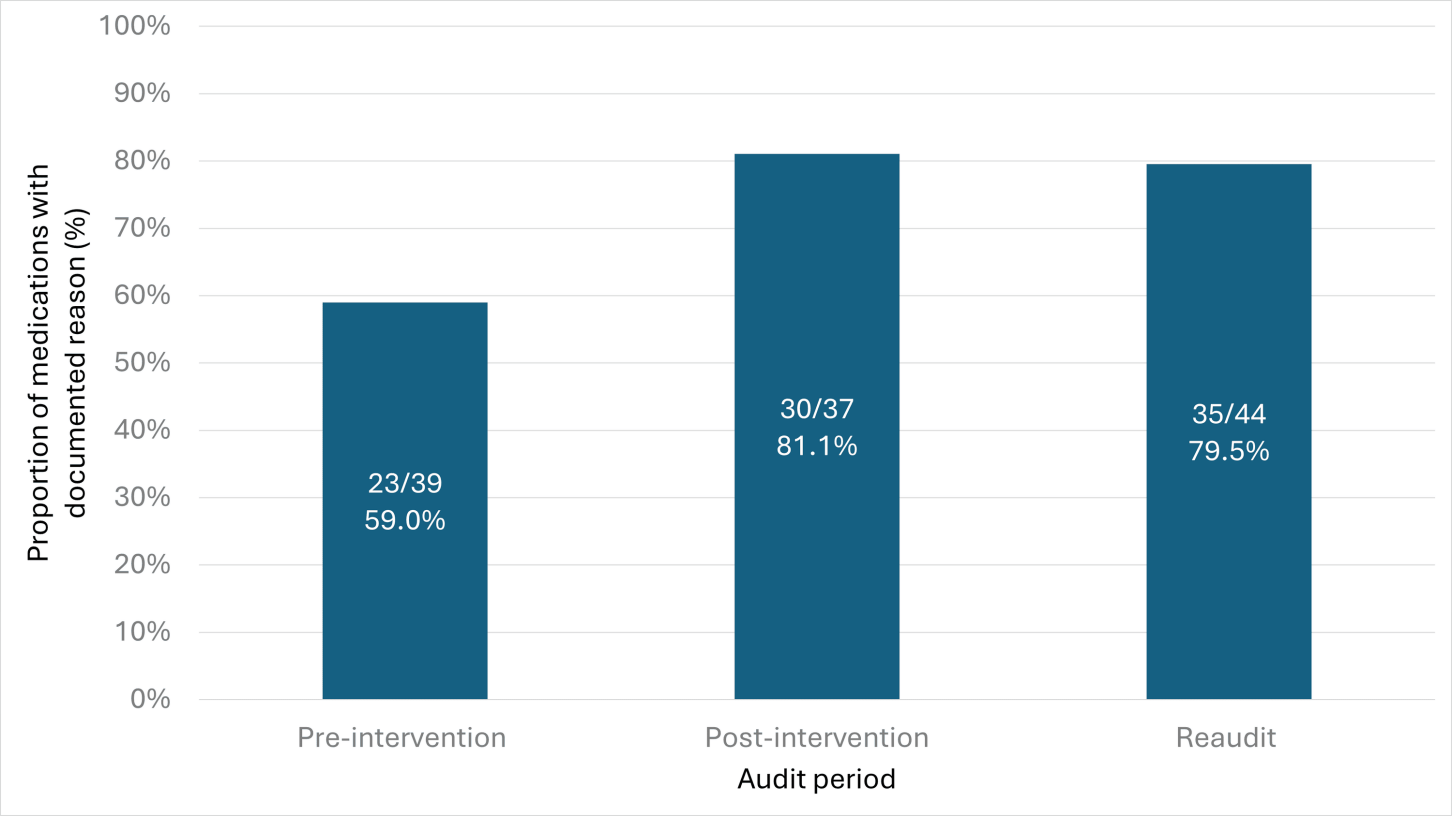
Proportion of withheld medications with a documented reason recorded in the clinical notes, drug chart, or medication reconciliation sheet across audit cycles

The proportion of withheld medications with a documented reason for withholding, recorded in any of the three possible locations (clinical notes, drug chart, or MR sheet), also showed a significant increase (Figure 2). This rose from 23 of 39 (59.0%) pre-intervention to 30 of 37 (81.1%) post-intervention (p = 0.036), and remained elevated at 35 of 44 (79.6%) during the reaudit (p = 0.041).

Withheld medication classes 

The most commonly withheld medication classes were diuretics (n = 27, 14.5%), angiotensin-converting enzyme inhibitors (ACEi) or angiotensin receptor blockers (ARBs; n = 24, 12.9%), and statins (n = 16, 8.6%) (Table 1). Cardiovascular medications represented the majority of withheld drugs, including diuretics, ACEi or ARBs, statins, calcium channel blockers (n = 13, 7.0%), beta-blockers (n = 6, 3.2%), and antiplatelets (n = 4, 2.2%). 

**Table 1:** Frequency and proportion of withheld medication classes This table lists all medication classes withheld at least once across the four audit periods (total withheld medications = 186). Values are presented as absolute frequency and percentage of the entire withheld medication sample. ACEi: angiotensin‑converting enzyme inhibitor, ARBs: angiotensin receptor blockers, DMARDs:  disease‑modifying antirheumatic drugs, NMDA: N‑methyl‑D‑aspartate.

Medication class	Frequency (n)	Proportion of all withheld medications
Diuretics	27	14.5%
ACEi and ARBs	24	12.9%
Statins	16	8.6%
Calcium channel blockers	13	7.0%
Oral hypoglycemic agents	12	6.5%
Vitamins/supplements	11	5.9%
Proton pump inhibitors	7	3.8%
Beta-blockers	6	3.2%
Direct oral anticoagulants	6	3.2%
Chemotherapy/immunotherapy	5	2.7%
Antiplatelets	4	2.2%
Laxatives	4	2.2%
Neuropathic agents	4	2.2%
Opiates	4	2.2%
Topical creams/ointments	4	2.2%
Antibiotics	3	1.6%
Anti-diarrhoeals	3	1.6%
Anti-inflammatories	3	1.6%
Inhalers	3	1.6%
Insulin	3	1.6%
Paracetamol	3	1.6%
Alpha-blockers	2	1.1%
Antacids	2	1.1%
Anti-anginals	2	1.1%
Antidepressants	2	1.1%
DMARDs	2	1.1%
Steroids	2	1.1%
Xanthine oxidase inhibitors	1	0.5%
Antiemetics	1	0.5%
Antipsychotics	1	0.5%
Antispasmodics	1	0.5%
Bisphosphonates	1	0.5%
Benzodiazepines and "Z-drugs"	1	0.5%
Glycosides	1	0.5%
NMDA receptor antagonist	1	0.5%
Phosphodiesterase-5 inhibitors	1	0.5%

Documented reasons for withholding medications 

The most frequently documented reasons for withholding medications were acute kidney injury (AKI) or dehydration (n = 39, 29.8%), hypotension (n = 15, 11.5%), and the medication being no longer clinically indicated (n = 15, 11.5%) (Table 2).

**Table 2:** Documented reasons for withholding medications This table summarizes the documented clinical reasons for withholding medications across all audit periods (total n = 131 documented reasons). "No longer indicated" refers to medications withheld due to clinical judgment that ongoing therapy was unnecessary. "Bleeding risk" includes patients with active or potential bleeding. "Possible adverse effect" indicates that the medication was withheld due to concerns that it was causing an adverse effect. IV: intravenous.

Documented reason	Frequency (n)	Proportion of all withholding reasons
Acute kidney injury/dehydration	39	29.8%
Hypotension	15	11.5%
No longer indicated	15	11.5%
Change to IV formulation	10	7.6%
Bleeding risk	7	5.3%
Infection	7	5.3%
Electrolyte abnormality	6	4.6%
No oral route available	6	4.6%
Hypoglycemia	5	3.8%
Possible adverse effect	5	3.8%
Polypharmacy/overdose	4	3.1%
Interaction with other medications	4	3.1%
Change to alternative medication	4	3.1%
Bradycardia	2	1.5%
Unable to confirm medication/medication dose	2	1.5%

## Discussion

Principal findings

This multicycle audit-feedback intervention led to marked improvements in two key medication safety processes: timely documentation of MR and clear recording of the rationale for withholding medications. Improvements were sustained over time, suggesting that the intervention had a lasting impact. These results suggest that simple, low-cost behavioral strategies can substantially enhance prescribing practices in a paper-based prescribing system.

Context within existing literature

Audit and feedback interventions are generally associated with absolute improvements in clinical practice of 4%-16% [10], particularly when feedback is timely, is individualized, and includes peer comparison. The 14%-17% improvement observed in this study lies at the higher end of this range, which may be due to the specific design of the intervention that incorporated personalized feedback, visible public recognition of high performers, and educational prompts in clinical areas.

Pharmacist-led MR is widely regarded as the gold standard and has been shown to reduce prescribing discrepancies, preventable adverse events, and hospital readmissions [7,11]. However, in many paper-based environments, limited pharmacy availability during admission means that MR initially falls to the admitting clinician. Our findings demonstrate that targeted feedback directed at prescribers alone can yield meaningful improvements before pharmacy review occurs.

The pattern of medications most commonly withheld (diuretics, ACEi or ARBs, statins, and calcium channel blockers) aligns with findings from previous studies in which cardiorenal agents represent the majority of withheld therapies [12]. The most frequently cited reasons for withholding, such as AKI, dehydration, and hypotension, are clinically appropriate and reflect a cautious approach to prescribing [13]. Nevertheless, even in the post-intervention period, in one of five cases, no reason for withholding was documented. This lack of documentation poses a risk of miscommunication among downstream care teams and with primary care clinicians after discharge, potentially leading to inappropriate continuation or cessation of previously withheld medications [14,15].

Strengths and limitations

Key strengths of this study include the use of prospectively defined process measures aligned with national MR standards [1,6]. The study included multiple time points of data collection, allowing assessment of both immediate and sustained impact. Additionally, it involved a pragmatic, low-resource intervention that can be implemented without additional training or resources. 

Limitations include the single-center setting in a paper-based environment, which may limit generalizability to digital systems where MR compliance is likely to be higher [16]. Follow-up extended only 10 days beyond the intervention; therefore, the longer-term sustainability of improvements remains unknown. The intervention was visible to clinicians, introducing the possibility of a Hawthorne effect. However, prescriber data were not collected during the reaudit week, suggesting that improvements were at least partially sustained in the absence of ongoing monitoring of individual clinicians. Furthermore, we did not collect feedback from clinicians regarding their perceptions of the intervention, its motivational value, or its impact on their workload. Finally, we did not assess the accuracy of MR entries, nor did we measure clinical outcomes such as adverse drug events, length of stay, or readmissions.

Implications for practice

In resource-constrained settings, clinician-centered feedback mechanisms can significantly improve MR documentation and the rationale for medication withholding. This intervention was implemented by existing staff without the requirement for additional training or prescribing systems. Aspects of the intervention, such as individualized statistics on MR compliance on admission, could feasibly be incorporated into existing systems of prescribing, especially in units using electronic systems. Until a point when interoperable medication records are adopted, hospitals could use a structured audit-feedback cycle with individualized and group feedback on MR completion compliance to aim for or maintain national benchmarks.

Improving the documentation of reasons for withholding medications remains a challenge in paper-based systems. Future developments in electronic prescribing should integrate mandatory fields for withholding rationale, either through free-text or drop-down options. Until such systems are universally adopted, iterative audit-feedback cycles may help embed a culture of accountability and completeness in documentation.

Future research

Future studies should investigate whether increased compliance with medication reconciliation and improved documentation of withheld medications translate into reductions in prescribing errors, adverse drug events, or overall healthcare costs. Further research is also warranted to evaluate the effectiveness of similar audit-feedback interventions in settings that use electronic prescribing systems. Finally, assessing the generalizability of this feedback model across different specialties and healthcare systems with varying admission processes would help determine its broader applicability.

## Conclusions

A targeted audit-feedback intervention directed at admitting clinicians significantly improved compliance with MR standards and documentation of withheld medications in a busy acute medical unit. While pharmacist involvement and electronic systems remain key components of medication safety, this study demonstrates that low-cost, clinician-led behavioral strategies can produce meaningful improvements in a short time frame and move practice closer to national safety benchmarks. These findings highlight the value of motivating admitting clinicians through structured feedback and peer comparison. Sustained implementation of similar interventions may help foster a culture of accountability and safe prescribing, particularly in resource-constrained, paper-based settings.
